# A pH-Responsive Drug Delivery System Based on Conjugated Polymer for Effective Synergistic Chemo-/Photodynamic Therapy

**DOI:** 10.3390/molecules28010399

**Published:** 2023-01-03

**Authors:** Chen Zhang, Qiong Yuan, Ziqi Zhang, Yanli Tang

**Affiliations:** Key Laboratory of Analytical Chemistry for Life Science of Shaanxi Province, School of Chemistry and Chemical Engineering, Shaanxi Normal University, Xi’an 710119, China

**Keywords:** conjugated polymer, drug release, photodynamic therapy, chemotherapy, antitumor treatment, synergistic therapy

## Abstract

Stimuli-responsive drug release and photodynamic therapy (PDT) have aroused extensive attention for their enormous potential in antitumor treatment. pH-responsive drug delivery systems (PFE-DOX-1 and PFE-DOX-2) based on water-soluble conjugated polymers were constructed in this work for high-performance synergistic chemo-/PDT therapy, in which the anticancer drug doxorubicin (DOX) is covalently attached to the side chains of the conjugated polymers via acid-labile imine and acylhydrazone bonds. Concurrently, the intense fluorescence of poly(fluorene-co-ethynylene) (PFE) is effectively quenched due to the energy/electron transfer (ET) between the PFE-conjugated backbone and DOX. Effective pH-responsive drug release from PFE-DOX-2 is achieved by the cleavage of acylhydrazone linkages in the acidic tumor intracellular microenvironment. Additionally, the drug release process can be monitored by the recovered fluorescence of conjugated polymers. Furthermore, the conjugated polymers can produce reactive oxygen species (ROS) under light irradiation after drug release in an acidic environment, which prevents possible phototoxicity to normal tissues. It is noted that PFE-DOX-2 demonstrates remarkable antitumor cell performance, which is attributed to its efficient cell uptake and powerful synergistic chemo-/PDT therapeutic effectiveness. This report thus provides a promising strategy for in vivo anticancer treatment with the construction of a stimuli-responsive multifunctional drug delivery system.

## 1. Introduction

Cancer, caused by abnormal cell proliferation and differentiation, has become one of the main factors threatening human health and reducing the quality of people’s lives [1]. It is thus of immense significance to explore up-to-date cancer diagnostic and treatment strategies, which are ongoing challenges for researchers in related fields [2,3]. Recently, photodynamic therapy (PDT) has provided a promising perspective for anticancer treatments [4,5,6]. Compared with conventional therapy methods, PDT possesses unique advantages, such as high spatial selectivity, negligible system toxicity and the prevention of multidrug resistance [7,8,9,10]. In the non-invasive PDT process, a photosensitizer (PS) plays a key role in the light-induced generation of cytotoxic reactive oxygen species (ROS) to eliminate cancer cells or pathogenic bacteria [11,12]. Generally, an ideal PS is expected to possess the necessary virtues, including a straightforward synthetic approach, high chemical purity, excellent biocompatibility and water solubility [13]. In recent years, a series of PSs have been fabricated, greatly facilitating the development of PDT techniques [14,15,16,17,18]. Moreover, dual or multi-modal treatment strategies with stimuli-responsive drug delivery have demonstrated satisfactory anticancer performance, such as PDT combined with chemotherapy [19,20,21,22]. As attractive candidates to be deployed as PDT reagents, conjugated polymers with excellent optical characteristics and modifiable structures can realize multiple functions through a deliberate design and elaborate synthesis to meet the demands of contemporary biomedical applications [23,24]. Conjugated polymers are an important category of organic materials, bearing large π-conjugated backbones and functional side chains. The transport of delocalized electrons along the conjugated framework enables the energy absorbed by the backbone to be converted into optical signals through electronic transitions, thereby endowing the conjugated polymers with outstanding light-harvesting capabilities and high fluorescence quantum yields [25,26,27,28]. In addition, hydrophilic groups can be introduced to the side chains to obtain water-soluble conjugated polymers for biological applications [24,29,30,31]. More importantly, conjugated polymers can generate ROS under light irradiation. Thus, conjugated polymers with excellent optical properties demonstrate good water solubility and biocompatibility, as well as negligible systemic toxicity, leading to their broad biomedical applications, including biosensing, cell imaging, and antibacterial and anticancer treatments. At present, most conjugated polymers used as PSs are physically mixed with drugs to construct chemo-/PDT dual-modal therapy platforms [32,33]. However, the uncertain drug leakage may cause extra damage to normal tissues and weaken the synergistic antitumor efficiency [34,35].

**Scheme 1:**
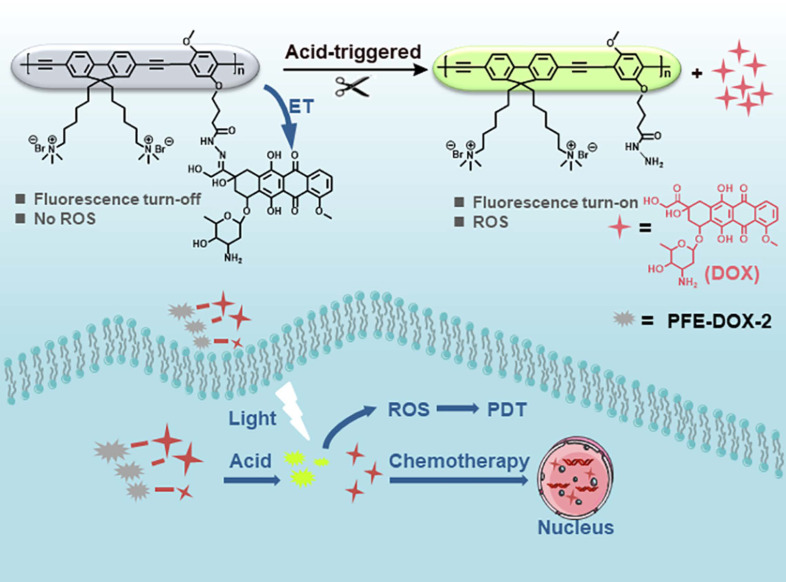
Schematic diagram of pH-responsive drug release and the mechanism of synergistic chemo-/PDT antitumor therapy.

Herein, a pH-responsive drug delivery system based on a conjugated polymer was constructed for effective synergistic chemo-/PDT antitumor therapy (Scheme 1). An anticancer drug, DOX, is covalently attached to the side chain of the polymer via the acid-sensitive acylhydrazone bond in PFE-DOX-2, which can improve the stability of the drug delivery system, preventing premature drug leakage. The fluorescence of the poly(fluorene-co-ethynylene) (PFE) backbone is effectively quenched by the energy/electron transfer (ET) between DOX and the conjugated backbone. Subsequently, the acidic tumor intracellular microenvironment can induce the breakage of acylhydrazone linkers, leading to drug release for chemotherapy. Additionally, the process of drug release can be monitored by measuring the recovery of the fluorescence intensity. Importantly, the generation of ROS can be triggered under light irradiation to realize synergistic chemo-/PDT antitumor therapy. This pH-responsive drug release system demonstrates excellent anticancer cell performance, thereby providing a feasible strategy for the construction of multifunctional stimuli-responsive drug delivery platforms.

## 2. Results and Discussion

### 2.1. Organic Synthesis

**Figure 1:**
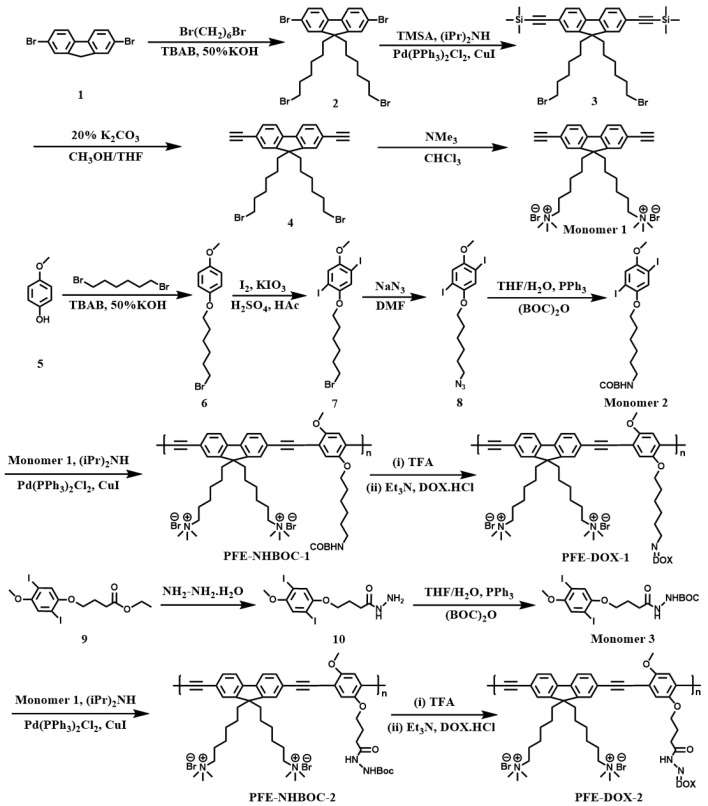
The synthetic routes of monomer 1, monomer 2, monomer 3, PFE-NHBOC-1, PFE-NHBOC-2, PFE-DOX-1 and PFE-DOX-2.

The synthetic routes of intermediates, monomers and conjugated polymers are shown in Figure 1. Monomer 1 and monomer 2 were synthesized according to our previous reports [36,37]. The conjugated polymers PFE-NHBOC-1 and PFE-NHBOC-2 were prepared via the Sonogashira coupling reaction between monomer 1 and monomer 2 or monomer 3, respectively. Then, the N-BOC protective groups of PFE-NHBOC-1 and PFE-NHBOC-2 were removed by trifluoroacetic acid (TFA). The imine bond and acylhydrazone bond were formed by the reaction between the carbonyl group of DOX and the amino groups on the conjugated polymers, respectively [38,39,40]. The detailed synthetic procedures of monomer 3, PFE-NHBOC-1, PFE-NHBOC-2, PFE-DOX-1 and PFE-DOX-2 are depicted in the Experimental Section of Supporting Information [41,42]. Additionally, the introduction of quaternary ammonium groups to the side chains of the conjugated polymers leads to their satisfactory water solubility.

### 2.2. Optical Properties of Conjugated Polymers

**Figure 2:**
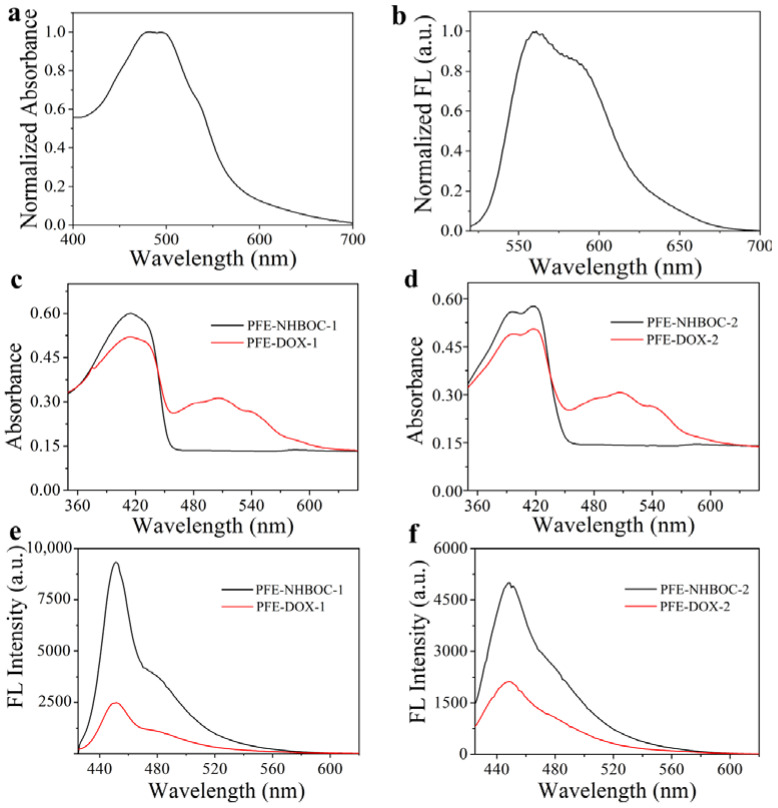
(**a**) Normalized absorption spectrum and (**b**) normalized fluorescence spectrum of DOX. Absorption spectra of conjugated polymers (**c**) PFE-NHBOC-1 and (**d**) PFE-NHBOC-2 before and after DOX modification. Fluorescence spectra of (**e**) PFE-NHBOC-1 and (**f**) PFE-NHBOC-2 before and after DOX modification.

The absorption and fluorescence spectra of PFE-NHBOC-1 and PFE-NHBOC-2 are illustrated in Figure 2. Owing to the same conjugated backbones of PFE-NHBOC-1 and PFE-NHBOC-2, these two polymers display similar absorption and emission bands centered at 415 nm and 450 nm, respectively. The conjugated polymers PFE-DOX-1 and PFE-DOX-2 were obtained by attaching DOX to PFE-NHBOC-1 and PFE-NHBOC-2 through imine and acylhydrazone bonds, respectively. Figure 2a,b show the absorption and emission spectra of DOX, respectively. As shown in Figure 2c,d, the characteristic absorption peak of DOX at around 480 nm was found in the absorption spectra of both PFE-DOX-1 and PFE-DOX-2, which indicated that DOX was covalently linked to the side chains of the conjugated polymers since the free drug DOX had been removed during the synthetic process by adequate dialysis. Figure 2e,f show that compared to their precursors, PFE-NHBOC-1 and PFE-NHBOC-2, the fluorescence of PFE-DOX-1 and PFE-DOX-2 was effectively quenched due to ET between DOX and PFE backbones. These results further proved the successful introduction of DOX to the side chains of the conjugated polymers.

### 2.3. ROS Generation In Vitro

Generally, PDT processes are classified into type I and type II pathways according to the principle of the photochemical reactions that produce ROS. Upon light irradiation, PSs are excited from the ground singlet state to the excited singlet state, followed by the formation of the excited triplet state (T_n_) through intersystem crossing (ISC). In the type I PDT process, PSs in the T_1_ state participate in the electron transfer and react with triplet oxygen (^3^O_2_) or a biological substrate to generate superoxide anions and hydroxyl radicals, respectively, whilst ^3^O_2_ is converted to ^1^O_2_ due to the energy transfer between T_1_-state PSs and the surrounding ^3^O_2_ in the type II PDT process. Thus, most PSs can produce cytotoxic ^1^O_2_ through an oxygen-dependent type II pathway, while few PSs are employed to generate ROS under hypoxic conditions [43,44].

**Figure 3:**
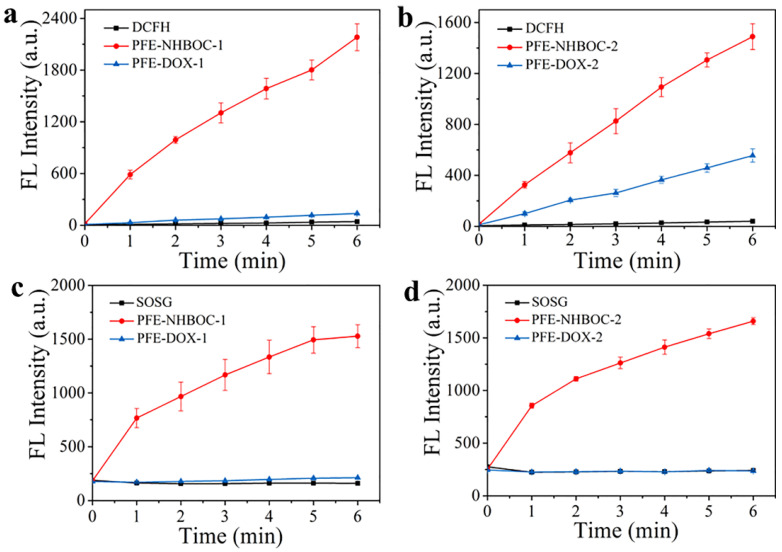
Fluorescence intensity of DCFH at 525 nm in the presence of (**a**) PFE-NHBOC-1 and PFE-DOX-1 and (**b**) PFE-NHBOC-2 and PFE-DOX-2 under white-light irradiation (5 mW cm^−2^) for 0–6 min: [polymer] = 1 μM; [DCFH] = 40 μM; λ_ex_ = 488 nm. Fluorescence intensity of SOSG at 525 nm in the presence of (**c**) PFE-NHBOC-1 and PFE-DOX-1 and (**d**) PFE-NHBOC-2 and PFE-DOX-2 under white-light irradiation (5 mW cm^−2^) for 0–6 min: [polymer] = 1 μM; [SOSG] = 1 μM; λ_ex_ = 504 nm.

The ROS generation ability of the conjugated polymers PFE-NHBOC-1, PFE-NHBOC-2, PFE-DOX-1 and PFE-DOX-2 was investigated by using ROS-sensitive 2′,7′-dichlorodihydrofluorescein diacetate (DCFH-DA) as an indicator. DCFH-DA can transform into non-fluorescence DCFH under alkaline conditions, which can generate DCF with intensive fluorescence after being oxidized by ROS. Hence, the ROS production performance can be reflected by the fluorescence intensity of DCF (λ_ex_ = 488 nm, λ_em_ = 525 nm). Under white-light irradiation, PFE-NHBOC-1 and PFE-NHBOC-2 displayed extremely high ROS production with a 51.9-fold and 37.2-fold enhancement of fluorescence intensity, respectively, as compared with that of the control DCFH group. However, the corresponding DOX-attached polymers PFE-DOX-1 and PFE-DOX-2 only gave weak emission peaks at 525 nm, which showed a 3.2-fold and 13.9-fold intensity enhancement compared to that of the control group (Figure 3a,b). This notable decrease in the ROS generation ability is ascribed to the fluorescence-quenching performance of DOX covalently linked to the side chains of the conjugated backbones, which can prevent possible phototoxicity to normal tissues when used for in vivo antitumor therapy.

^1^O_2_ is a vital cytotoxic ROS, demonstrating powerful damaging effects on tumor cells. The ^1^O_2_ generation capability of these four conjugated polymers was thus explored by employing non-luminescence singlet oxygen sensor green (SOSG) as the sensitive probe, which can display strong fluorescence emission at 525 nm (λ_ex_ = 504 nm) after its interaction with ^1^O_2_. In comparison with the polymer-free control group, the fluorescence intensity of SOSG at 525 nm was increased by 9.4 and 6.9 times after white-light irradiation in the presence of PFE-NHBOC-1 and PFE-NHBOC-2, respectively, whereas there was a negligible change in the fluorescence intensity at 525 nm in the PFE-DOX-1 and PFE-DOX-2 groups under the same testing conditions (Figure 3c,d). From the discussion above, the ROS generated from PFE-NHBOC-1 and PFE-NHBOC-2 are attributed to ^1^O_2_ produced by the type II pathway, accompanied by the generation of other ROS that relies on the type I mechanism. Further investigations are needed to elucidate the detailed mechanism of ROS production in these polymers. These results demonstrate the remarkable ROS generation ability of PFE-NHBOC-1 and PFE-NHBOC-2, which can be deployed as effective PDT agents for antitumor therapy.

### 2.4. Drug Release In Vitro

**Figure 4:**
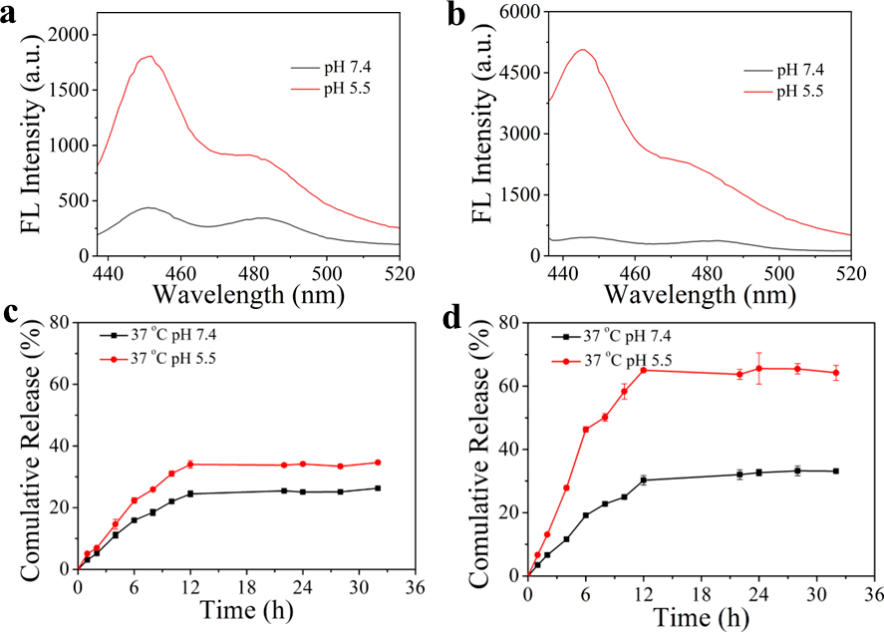
The fluorescence recovery effect of (**a**) PFE-DOX-1 and (**b**) PFE-DOX-2 after the reaction in PBS buffer with different pH values. DOX release curves of (**c**) PFE-DOX-1 and (**d**) PFE-DOX-2 in PBS buffer with different pH values.

The fluorescence spectra of PFE-DOX-1 and PFE-DOX-2 were recorded before and after the drug release process in order to determine the fluorescence recovery effect of these polymers induced by drug release. These polymers were reacted in PBS buffer with the same ionic strength but different pH values, followed by the detection of their fluorescence intensity. The drug loading rates of PFE-DOX-1 and PFE-DOX-2 are 14.0% and 12.9%, respectively. As illustrated in Figure 4a,b, the emission intensity of the conjugated backbone at 450 nm showed a distinct increase for both the PFE-DOX-1 and PFE-DOX-2 solutions at pH 5.5 after the drug release process, which was much higher than that of the polymer solutions at pH 7.4. Thus, this pH-dependent fluorescence recovery in PFE-DOX-1 and PFE-DOX-2 enables them to realize the real-time monitoring of drug release. In particular, the fluorescence recovery performance of PFE-DOX-2 after DOX release in acidic conditions is superior to that of PFE-DOX-1, which may result from the lower molecular weight and the better water solubility of PFE-DOX-2, leading to the more efficient acid hydrolysis of the acylhydrazone bond.

Subsequently, the pH-responsive drug release from PFE-DOX-1 and PFE-DOX-2 was investigated by dialysis, utilizing PBS buffer with the same ionic strength but different pH values as the dialysis medium. As shown in Figure 4c,d, the drug release rates of PFE-DOX-1 and PFE-DOX-2 at pH 7.4 were 26% and 32%, which increased to 34% and 66% at pH 5.5, respectively. Hence, the specific drug release from PFE-DOX-1 and PFE-DOX-2 under acidic conditions is realized by the acidolysis of imine and acylhydrazone bonds, respectively (Figure 4a,b).

### 2.5. Cellular Uptake and Drug Release

The uptake of the DOX-attached polymer by tumor cells and drug release in cells were investigated by employing confocal laser scanning microscopy (CLSM). Since the drug release effect of PFE-DOX-2 in vitro is superior to that of PFE-DOX-1, the related performance of PFE-DOX-2 was studied in-depth in the following experiments. As shown in Figure S1, after being incubated with PFE-DOX-2 for 2 h and 6 h, the green fluorescence of PFE-DOX-2 and the red fluorescence of DOX were both observed in MCF-7 cells, indicating the effective uptake of hydrophobic DOX by the tumor cells, employing the water-soluble conjugated skeleton as the drug delivery platform. The old medium was then replaced with the fresh medium, followed by the incubation of MCF-7 cells for 8 h, 12 h and 24 h to evaluate drug release from and fluorescence changes in the ingested PFE-DOX-2 in tumor cells. With the extension of the incubation time, more and more green fluorescence of the PFE backbone was observed, indicating the fracture of acylhydrazone bonds and efficient drug release from PFE-DOX-2 triggered by the acidic microenvironment of MCF-7 cells. These results demonstrate intracellular drug release from PFE-DOX-2, which can be monitored by measuring the change in the polymer’s fluorescence intensity in cells.

### 2.6. ROS Generation in Cells

**Figure 5:**
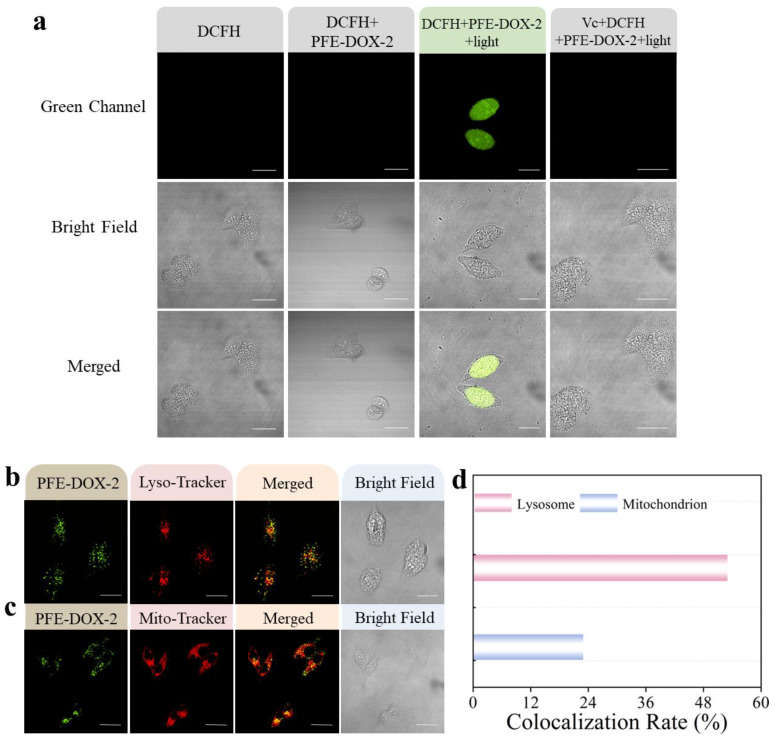
(**a**) Detection of ROS generation ability of PFE-DOX-2 in MCF-7 cells with or without light irradiation (90 mW cm^−2^) for 15 min. The fluorescence signal was collected in the range of 500–600 nm (λ_ex_: 488 nm) for DCFH. [PFE-DOX-2] = 1 μM, [DCFH] = 20 μM, [Vc] = 100 μM. CLSM images of MCF-7 cells treated with PFE-DOX-2 (1 μM) and stained with (**b**) Lyso-Tracker red DND 99 and (**c**) Mito-Tracker Deep Red FM. The fluorescence signal was collected in the range of 410–460 nm (λ_ex_: 405 nm) for PFE-DOX-2, 575–620 nm (λ_ex_: 559 nm) for Lyso-Tracker red DND 99, and 650–700 nm (λ_ex_: 635 nm) for Mito-Tracker Deep Red FM, respectively. Scale bar is 40 μm. (**d**) Pearson’s coefficient of PFE-DOX-2 and Lyso-Tracker red DND 99 or Mito-Tracker Deep Red FM in MCF-7 cells.

Apart from drug release for chemotherapy, ROS production from the as-prepared polymer in tumor cells also plays a crucial role in synergistic chemo-/PDT therapy. The intracellular ROS generation capability of PFE-DOX-2 was investigated by using DCFH-DA as an indicator. MCF-7 cells were first incubated in the medium containing PFE-DOX-2, and then the cells were treated with DCFH-DA to evaluate the ROS production effect by detecting the fluorescence intensity. As shown in Figure 5a, there was no fluorescence observed in cells incubated with only DCFH-DA. However, bright fluorescence in the green channel was observed after the white-light irradiation of cells that were treated with both PFE-DOX-2 and DCFH-DA, whereas no obvious fluorescence was found in the cell group with the absence of light irradiation. Furthermore, the green fluorescence vanished after the addition of Vitamin C (Vc) into the system, since Vc can effectively remove ROS as an inhibitor. Therefore, the intracellular ROS production ability of PFE-DOX-2 has been confirmed, contributing to synergistic chemo-/PDT therapy. In addition, the distribution of PFE-DOX-2 after its cellular uptake was investigated, for which the lysosomes and mitochondria were stained with Lyso-Tracker red DND 99 and Mito-Tracker Deep Red FM, respectively. As illustrated in Figure 5b,c, the fluorescence of PFE-DOX-2 in the green channel showed a better overlap with the fluorescence of lysosomes than mitochondria after incubating the cells with PFE-DOX-2 for 4 h. The Pearson coefficient of PFE-DOX-2 and lysosomes is 53%, which is much higher than that of mitochondria (23%), indicating that PFE-DOX-2 mainly accumulates in lysosomes upon its cellular uptake (Figure 5d).

### 2.7. Synergistic Chemo-/PDT Therapy

**Figure 6:**
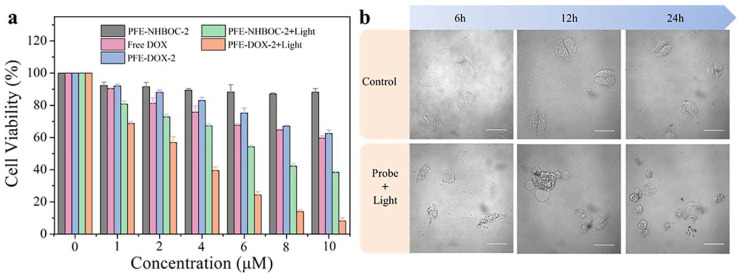
(**a**) Cytotoxicity of PFE-NHBOC-2, PFE-DOX-2 and free DOX against MCF-7 cells without or with light irradiation (25 mW cm^−2^) for 30 min. (**b**) The morphological changes in MCF-7 cells incubated with PFE-DOX-2 and treated with light irradiation over time. Scale bar is 40 μm.

The cytotoxicity of PFE-NHBOC-2 and PFE-DOX-2 towards MCF-7 cells was investigated by using a typical MTT assay. As illustrated in Figure 6a, the viability of MCF-7 cells remains above 90% after treating the cells with PFE-NHBOC-2 at 10 μM, suggesting the negligible dark cytotoxicity and excellent biocompatibility of PFE-NHBOC-2. Conversely, the dark cytotoxicity of PFE-DOX-2 to MCF-7 cells was distinctly enhanced with the increase in the polymer concentration, and cell viability was reduced to 69% when the cells were incubated with PFE-DOX-2 at 10 μM, indicating the effective drug release from the polymer in tumor cells without light irradiation.

After white-light irradiation (25 mW cm^−2^, 30 min), PFE-NHBOC-2 displayed significant cytotoxicity against MCF-7 cells, and cell viability was reduced to 38% when the concentration of PFE-NHBOC-2 was increased to 10 μM. Noteworthily, the viability of cells co-incubated with PFE-DOX-2 was impressively reduced under white-light irradiation, which was as low as 10% with the concentration of PFE-DOX-2 at 10 μM. The cytotoxicity of PFE-DOX-2 under light irradiation is higher than that of light-irradiated PFE-NHBOC-2 and free DOX at the same concentrations, proving the excellent synergistic chemo-/PDT therapeutic effect of PFE-DOX-2. Consequently, the satisfactory synergistic therapeutic performance of PFE-DOX-2 is derived from two indispensable elements: the efficient pH-responsive drug release and sufficient light-triggered ROS generation. The synergistic therapeutic effect of PFE-DOX-2 can also be reflected by the cell morphologies before and after the synergistic therapy process. As shown in Figure 6b, well-shaped MCF-7 cells were observed in the blank group with the increase in the incubation time, whereas the generated ROS in the polymer-treated group continued to damage the cancer cells. Damaged cells were found in the cell group treated with PFE-DOX-2 and light irradiation after 6 h, while a large number of cells were necrotic after 12 h. After the incubation of cells for 24 h, the number of necrotic cells was significantly increased. All of these results demonstrate the powerful synergistic therapeutic effect of PFE-DOX-2 on cancer cells.

## 3. Materials and Methods

### 3.1. Experimental Reagents and Instruments

The chemical reagents utilized in the organic synthesis and analytical experiments were purchased from J&K Scientific Ltd. (Beijing, China), Aladdin Biochemical Technology Co., Ltd. (Shanghai, China), Sinopharm Chemical Reagent Co., Ltd. (Shanghai, China), and Sangon Biotech Co., Ltd. (Shanghai, China). All reagents were analytically pure and used directly without purification unless otherwise specified. The water used in organic synthesis and analytical experiments was distilled water and ultrapure water, respectively. For cell experiments, the DMEM medium, fetal bovine serum (FBS) and trypsin were purchased from Thermo Fisher Scientific Co., Ltd. (Shanghai, China). Lyso-Tracker DND 99 and Mito-Tracker Deep Red FM were purchased from Yeasen Biotechnology (Shanghai, China).

The NMR spectra of the intermediates, monomers and polymers were recorded using 300 AVANCF 300MHz, Bruker Ascend TM 400 or AVANCE III 600 (Bruker Corporation, Tokyo, Japan). A Bruker maXis II mass spectrometer (Bruker Corporation, Bremen, Germany) was employed to measure the mass spectra. The molecular weights of the conjugated polymers in this report were determined using a laser light scattering gel chromatography system (Viscotek TM, Malvern Corporation, Malvern, UK). The fluorescence and UV-vis absorption spectra were recorded on a Hitachi F-7000 spectrophotometer (Tokyo, Japan) and SHIMADZU UV-2600 spectrophotometer (Kyoto, Japan), respectively. A microplate reader Spectramax M5 (Molecular Devices, San Jose, CA, USA) was utilized to investigate the absorbance in the cytotoxicity test. The CLSM images of cells were recorded with Olympus FV1200 (Tokyo, Japan).

### 3.2. ROS Generation In Vitro

DCFH-DA was utilized to detect the generation of ROS. The conjugated polymer solution was added to a DCFH solution (40 μM) to obtain a 2 mL polymer solution (1 μM) for the investigation, while there was only a 2 mL DCFH solution in control groups. The solutions were irradiated under white light (5 mW cm^−2^), and the fluorescence intensity of the above solutions at 525 nm was recorded at 0, 1, 2, 3, 4, 5 and 6 min with an excitation wavelength of 488 nm. SOSG was employed to evaluate the ^1^O_2_ production effect. The polymer solution was added to the SOSG stock solution to prepare the test solution (1 μM). Under white-light irradiation (5 mW cm^−2^), the fluorescence intensity of the solutions at 525 nm was recorded at 0, 1, 2, 3, 4, 5 and 6 min (λ_ex_ = 504 nm).

### 3.3. Drug Release In Vitro

Drug release from the conjugated polymers PFE-DOX-1 and PFE-DOX-2 in vitro was determined by dialysis (MWCO: 3500 Da) at 37 °C [30,45]. A 2.5 mL polymer solution was added to a dialysis bag and dialyzed in 30 mL of PBS buffer with a pH value of 5.5 or 7.4. After different time periods, 2 mL of dialysate was pipetted to record the fluorescence intensity of DOX at 560 nm. The amount of the released drug was calculated according to the emission standard curve of DOX. In the dialysis process, PFE-DOX-1 and PFE-DOX-2 cannot permeate through the dialysis membrane due to their high molecular weights; the fluorescence intensity changes in the dialysate at 560 nm are thus ascribed to DOX release from the conjugated polymers.

### 3.4. Cellular Uptake and Intracellular Drug Release

Cell experiments were performed using MCF-7 cells (human breast cancer cells) in Dulbecco’s modified Eagle medium (DMEM). Specifically, MCF-7 cells were incubated in confocal culture dishes for 12 h at 37 °C. Then, the previous medium was removed and replaced with fresh medium containing PFE-DOX-2 (1 μM). The cells were then cultured for another 2 h or 6 h and washed with PBS 3 times before CLSM imaging. To better observe drug release from the polymer in cells, the old medium in other cell groups was replaced with fresh medium again, and the cells were sequentially incubated for 8 h, 12 h or 24 h (there was no cellular uptake of the polymer, but intracellular drug release occurred in this process). Then, the previous medium was removed for CLSM imaging.

### 3.5. ROS Generation in Cells

MCF-7 cells were co-incubated with PFE-DOX-2 (1 μM) for 6 h, and then the polymer-containing medium was replaced with fresh medium. Sequentially, the cells were washed with PBS buffer and cultured in medium containing DCFH-DA (20 μM) for 30 min. Then, the cells were washed with PBS, followed by irradiation under white light (90 mW cm^−2^) for 15 min before CLSM imaging.

### 3.6. Synergistic Chemo-Photodynamic Therapy

The synergistic therapeutic effect of PFE-NHBOC-2, free DOX and PFE-DOX-2 on MCF-7 cells was investigated using the 3-(4,5-dimethylthiazol-2-yl)-2,5-diphenyltetrazolium bromide (MTT) assay. Suspended MCF-7 cells were seeded into 96-well plates and incubated for 12 h. After removing the previous medium, fresh medium containing PFE-NHBOC-2, free DOX or PFE-DOX-2 at various concentrations (0 μM, 1 μM, 2 μM, 4 μM, 6 μM, 8 μM and 10 μM) was added, and the cells were then incubated for 48 h. Subsequently, 10 μL of MTT solution (5 mg mL^−1^) was added to each well for incubation for another 4 h. Finally, the foregoing medium was replaced with DMSO (100 μL) for the measurement of the absorbance value of each well at 490 nm using a microplate reader. The antitumor efficacy of PFE-NHBOC-2 and PFE-DOX-2 under light irradiation was also investigated. The cells were first incubated in medium containing polymers at various concentrations for 6 h, followed by irradiation under white light (25 mW cm^−2^) for 30 min. The cells were then incubated for 42 h before the addition of MTT solution. The remaining operations were the same as those in the investigation of dark cytotoxicity.

## 4. Conclusions

In conclusion, a multifunctional pH-responsive drug delivery system based on a conjugated polymer has been successfully fabricated. The hydrophobic anticancer drug DOX is covalently attached to the water-soluble conjugated polymer via the acid-labile acylhydrazone bond, preventing premature drug leakage. The fluorescence of the conjugated backbone is effectively quenched due to the ET effect between DOX and the conjugated backbone. In addition, the utilization of an amphiphilic conjugated polymer skeleton as the drug delivery vehicle contributes to the uptake of the hydrophobic drug by cancer cells. The acidic microenvironment of cancer cells ensures the effective drug release induced by the breakage of the acylhydrazone bond, which results in the fluorescence recovery of the conjugated polymer, contributing to the monitoring of the drug release process and light-triggered PDT therapy. The conjugated polymer PFE-DOX-2 displayed remarkable synergistic chemo-/PDT therapeutic efficacy, which is superior to that of the individual therapy method. Hence, this pH-responsive drug delivery system realized acid-triggered and monitorable drug release, as well as a powerful synergistic chemo-/PDT therapeutic effect, thereby providing an exploitable approach for antitumor treatment.

## Data Availability

The data presented in this study are available on request from the corresponding author.

## Supplementary Materials

The following supporting information can be downloaded at: https://www.mdpi.com/article/10.3390/molecules28010399/s1. Figure S1: CLSM images of MCF-7 cells after incubation with PFE-DOX-2 (1 μM) for different times. Experimental section: Synthesis of monomer 3; Synthesis of PFE-NHBOC-1; Synthesis of PFE-DOX-1; Synthesis of PFE-NHBOC-2; Synthesis of PFE-DOX-2.

## Sample Availability

Samples of the compounds are available from the authors.
