# Supplementary material for: A pH-Responsive Drug Delivery System Based on Conjugated Polymer for Effective Synergistic Chemo-/Photodynamic Therapy
Source: Molecules. 2023 Jan 3;28(1):399. doi: 10.3390/molecules28010399 (PMC9823741; doi:10.3390/molecules28010399)
Supplement: Supplementary file 1 [file molecules-28-00399-s001.zip › molecules-2086509-supplementary.pdf]

Supporting Information

A pH-Responsive Drug Delivery System Based on Conjugated Polymer for Effective Synergistic Chemo-/Photodynamic Therapy

Chen Zhang, Qiong Yuan, Ziqi Zhang and Yanli Tang *

Key Laboratory of Analytical Chemistry for Life Science of Shaanxi Province, School of Chemistry and Chemical Engineering, Shaanxi Normal University, Xi’an 710119, China

* Correspondence: yltang@snnu.edu.cn

Table of Contents

1. Experimental section S3

2. Figure S1 ....S6

Experimental section

Synthesis of monomer 3.

The synthetic reaction from compound 9 to monomer 3 is carried out in one system, and compound 9 was synthesized previously by our group. Firstly, compound 9 (0.49g, 1 mmol) was added into methanol (10 mL), hydrazine hydrate (10 mL, 0.39 mol) was then dropwise added to the mixed solution, and the reaction solution was stirred overnight at room temperature. The resultant solution was extracted by dichloromethane and then dried with anhydrous magnesium sulfate. The organic solvent was removed to obtain the white solid product (compound 10). The prepared compound 10 was dissolved in THF (8 mL), following by the addition of di-tert-butyl dicarbonate (500 μL, 2 mmol), the mixed solution was stirred at room temperature for 12 h. The reaction solution was then extracted and dried by dichloromethane and magnesium sulfate, respectively. The white solid targeted product monomer 3 was obtained by silica gel column chromatography (356 mg, 60 %). ^{1}H NMR (300 MHz, DMSO-d_6) δ: 9.50 (s, 1 H), 8.60 (s, 1 H), 7.26 (s, 2 H), 3.99-3.90 (m, 2 H), 3.70 (s, 3 H), 2.27-2.19 (m, 2 H), 1.92-1.86 (m, 2 H), 1.33 (s, 9 H). ^{13}C NMR (151 MHz, $CDCl_3$) δ 155.33, 153.57, 123.19, 121.36, 86.23, 85.58, 81.92, 77.23, 77.02, 76.81, 68.73, 57.18, 30.39, 28.15, 24.77. HRMS (ESI): m/z: 598.9516 ($[M+Na]^+$).

Synthesis of PFE-NHBOC-1.

Monomer 1 (60 mg, 0.09 mmol). monomer 2 (52 mg, 0.09 mmol), diisopropylamine (0.5 mL), bis(triphenylphosphine)palladium(II) chloride (6 mg, 0.0075 mmol) and CuI (1.5 mg, 0.0075 mmol) were dissolved in DMF (2 mL) under nitrogen atmosphere. The

reaction solution was stirred under room temperature for 12 h. The cooled resultant solution was dialyzed for 4 days utilizing a membrane (MWCO: 3.5 kDa). Brown solid product was obtained after freeze drying (57 mg, 65%). ^{1}H NMR (600 MHz, DMSO-d_6) δ: 7.93-7.07 (m), 6.78 (s), 4.05-3.71 (m), 3.13 (d), 2.94 (br), 2.46 (s), 2.05 (br), 1.77-0.81 (m), 0.45 (br). GPC: M_n = 17281, M_w = 22501, PDI = 1.302.

Synthesis of PFE-DOX-1.

(1) Doxorubicin hydrochloride (DOX·HCl) (21 mg, 0.036 mmol) and triethylamine (30 μL) was added into DMSO (2 mL), the mixture was stirred for 2 h in dark to obtain DOX. (2) PFE-NHBOC-1 (30 mg, 0.024 mmol) was dissolved in methanol (5 mL), following by the addition of trifluoroacetic acid (90 μL, 1.2 mmol), the mixed solution was stirred overnight. Triethylamine (66 μL) was added into the reaction system after the trifluoroacetic acid was removed by rotary evaporation. After the mixture was stirred for 5 h, triethylamine and methanol were both dried to obtain the yellow solid product. (3) The yellow solid in step (2) was dissolved by DMSO (1 mL), following by the addition of the obtained solution in step (1) and triethylamine (50 μL). Reddish-brown liquid was obtained after the mixed solution was stirred at 50 °C for 24 h. The resultant solution was dialyzed for 4 days utilizing a membrane (MWCO: 3.5 kDa). Finally, the reddish-brown solid was obtained after freeze drying (23.8 mg, 70%). ^{1}H NMR (600 MHz, DMSO-d_6) δ: 7.87-7.43 (m), 7.20-7.05 (m), 6.78 (br), 3.98-3.53 (m), 2.90 (s), 2.15-1.81 (m), 1.66-0.46 (m). GPC: M_n = 16937, M_w = 21486, PDI = 1.268.

Synthesis of PFE-NHBOC-2.

PFE-NHBOC-2 was prepared by the similar synthetic strategies as PFE-NHBOC-1,

except that monomer 2 was replaced by monomer 3, following by the coupling reaction with monomer 1 to produce brown color solid (52.9 mg, 60%). ^{1}H NMR (600 MHz, DMSO-d_6) δ: 7.85-7.40 (m), 7.29-6.87 (m), 4.03-3.66 (m), 2.91 (d), 2.22-1.67 (m), 1.4 (d), 1.14-0.96 (m), 0.77-0.69 (m), 0.41 (br). GPC: M_n = 7010, M_w = 7835, PDI = 1.117.

Synthesis of PFE-DOX-2.

The preparation and processing methods of PFE-DOX-2 are similar to those of PFE-DOX-1, except that PFE-NHBOC-2 is involved in the related reactions to obtain reddish-brown solid (26.6 mg, 78 %). ^{1}H NMR (600 MHz, DMSO-d_6) δ: 8.22-7.03 (m), 4.04-3.70 (m), 2.87 (br), 2.31-2.21 (m), 2.01-1.84 (m), 1.39-1.25 (m), 1.15 (br), 0.95 (br), 0.8-0.75 (m). GPC: M_n = 11140, M_w = 12687, PDI = 1.138.

Figure S1

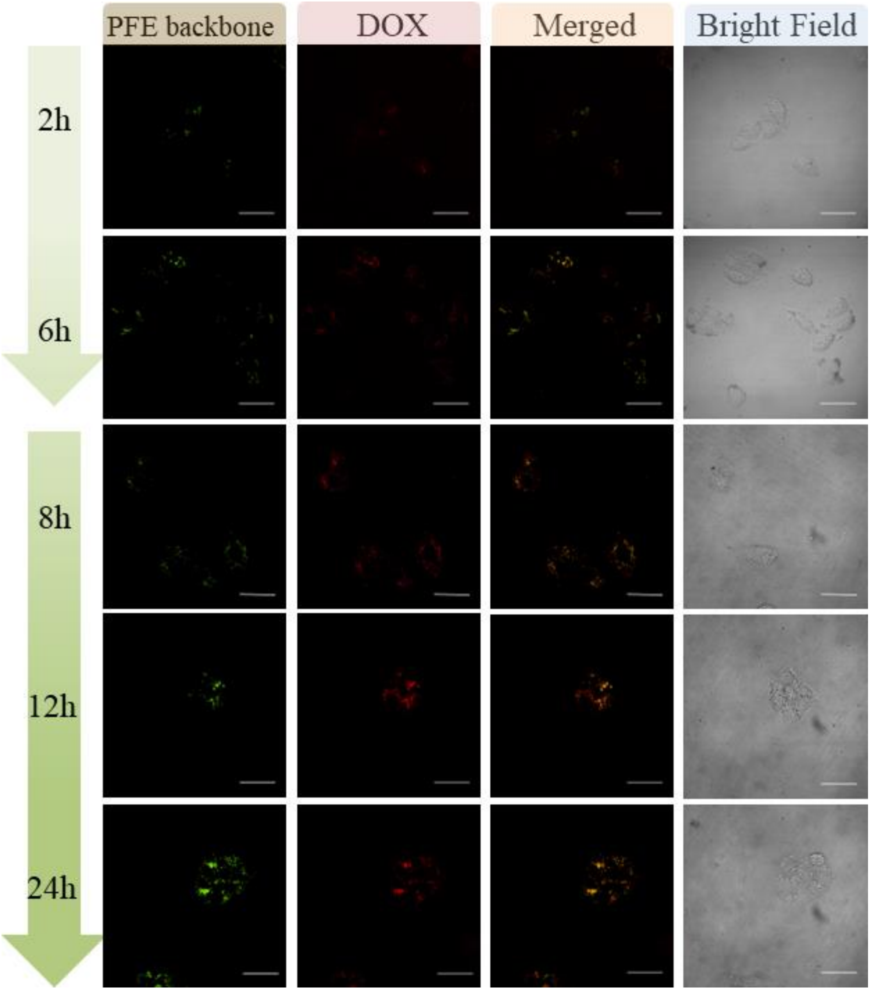

Figure S1. CLSM images of MCF-7 cells after incubation with PFE-DOX-2 (1 μM) for different times. The fluorescence signal was collected in the range of 410-460 nm (λ_{ex}: 405 nm) for PFE-DOX-2, 500-600 nm (λ_{ex}: 488 nm) for DOX, respectively. Scale bar is 40 μm.
